# Effect of Microbiota-Selenoprotein on Meat Selenium Content and Meat Quality of Broiler Chickens

**DOI:** 10.3390/ani10060981

**Published:** 2020-06-04

**Authors:** Dalia A. Mohamed, Awis Qurni Sazili, Loh Teck Chwen, Anjas Asmara Samsudin

**Affiliations:** 1 Department of Animal Science, Faculty of Agriculture, Universiti Putra Malaysia, Serdang 43400, Selangor, Malaysia; dalida983@yahoo.com (D.A.M.); awis@upm.edu.my (A.Q.S.); tcloh@upm.edu.my (L.T.C.); 2 Department of Animal Nutrition, Faculty of Animal Production, University of Khartoum, Khartoum 11111, Sudan

**Keywords:** selenium, bacteria, breast meat, antioxidant, hematology, carcass

## Abstract

**Simple Summary:**

Lack of selenium (Se) is a worldwide problem which leads to an increased exposure to various diseases in animals and humans, as well as decreased productive and reproductive performance of animals. Due to the health benefits of this element, it can be supplemented to humans as multimineral containing inorganic Se and *Saccharomyces* *cerevisiae* yeast, which mainly contains selenomethionine. On the other hand, selenium-containing food products—such as selenium-rich meat—can be considered as functional foods, which can be produced easily using organic selenium. This study states that Se-enriched breast meat with good antioxidant capacity can be produced using extracted bacterial selenoprotein.

**Abstract:**

Selenium (Se) is able to transform from inorganic to organic forms via many bacterial species. This feature is being considered for delivering more bioavailable selenium compounds such as selenocysteine and selenomethionine for human and animal diet. This study investigated the effects of bacterial selenoprotein versus inorganic Se on the carcass characteristics, breast meat selenium content, antioxidant status, and meat quality of broiler chickens. One hundred and eighty chicks were randomly allotted to five treatments of a basal diet supplemented with no Se, sodium selenite, *Enterobacter* *cloacae* Selenium (ADS1-Se), *Klebsiella* *pneumoniae*-Selenium (ADS2-Se), and *Stenotrophomonas* *maltophilia*-Selenium (ADS18-Se). The results showed that bacterial selenoprotein has the ability to deposit more Se in the breast meat compared to sodium selenite. Both Se sources reduced breast meat drip loss, cooking loss, shear force, and 2-thiobarbituric acid reactive substances (TBARS) significantly. It also increased total antioxidant (TAC) and glutathione peroxidase (GSH-Px) in comparison with the negative control. The highest activity of (GSH-Px), catalase (CAT), and superoxide dismutase (SOD) was found in bacterial selenoprotein. In conclusion, bacterial selenoprotein is more efficient than sodium selenite in increasing the breast meat Se deposition and oxidative capacity of broiler chickens. Therefore, it can be effectively used to produce Se-rich meat as a functional food.

## 1. Introduction

Meat quality and stability are affected mainly by the lipid peroxidation which is related to the production of reactive free radicals. Excessive reactive free radicals will reduce meat sensory traits and nutritional values. In living organisms, antioxidant processes protect the body cells from the harmful effects of free radicals [1]. The supplementation of Se is necessary for maintaining the high performance of broiler chickens [2], a minimum level for selenium supplementation of broiler chickens is 0.15 mg/kg depending on the variation of this mineral concentration in animal feed [3]. Moreover, the efficiency of Se supplementation depends on the dietary form [4]. The bioavailability of selenium is linked to its physical form. Currently, sodium selenite is the most commonly used selenium source in animal feeds; however, it has some disadvantages of lower availability, as well as potential toxicity at higher concentration. It can also produce some free radicals and cause pro-oxidation effects. On the other hand, organic forms like selenium-enriched yeast and selenomethionine are utilized in many countries as a safer and better source of Se in animal feed. These Se forms showed high bioavailability and ability to improve meat quality and oxidative stability of broiler chickens, as well as production of Se-enriched meat [5]. According to Bakhshalinejad et al. [6] supplementation of organic sources of Se (Se-yeast, selenomethionine, and nano-Se) resulted in a better meat quality compared with the inorganic source (sodium selenite) in broiler chickens. Moreover, dietary intake of 0.3 g/kg Se-yeast, Se-methionine, and nano-Se showed lower levels of shear force and cooking loss with high level of GSH-Px of breast muscles in local Chinese Subei chickens compared to the sodium selenite [7].

On the other hand, Se supplementation in the form of probiotics bacteria and bacterial extract has received more consideration in the last years [8], a wide range of selenium enriched bacteria were identified as organic source of selenium since they are able to uptake inorganic Se and accumulate it in their cells in the form of selenoprotein [9]. Previously, we stated that some organic Se-enriched bacteria identified as *Enterobacter cloacae*, (ADS1), *Klebsiella pneumoniae* (ADS2), and *Stenotrophomonas maltophilia* (ADS18), were showed high ability to accumulate around 50% of their absorbed Se as selenoproteins in their cells, and their extracted selenoprotein showed high Se concentrations with moderate antioxidant properties, which can be considered as a potential source of organic Se [10,11]. Mineral functional food showed a beneficial effect on human health and nutritional status, it decreasing the minerals deficiency issues and prevent humans from various diseases. Hence, this current study attempted to investigate the ability of producing Se-enriched meat using various bacterial sources of Se as an alternative organic Se supplement, as well as to establish their effect on the carcass characteristics, meat quality, and meat antioxidant in broiler chicken.

## 2. Materials and Methods

### 2.1. Extraction of Bacterial Selenium Content

*Enterobacter cloacae* (ADS1), *Klebsiella pneumoniae* (ADS2), and *Stenotrophomonas maltophilia* (ADS18) were isolated from rumen fluid and hot spring water according to its high seleno-amino acids mainly Se-meth. The stock cultures were prepared at the Laboratory of Microbiology, Department of Animal Science at the Faculty of Agriculture, Universiti Putra Malaysia (UPM) according to the method described by Dalia et al. [11]. The 30% glycerol stock culture of each strain was revived three times in nutrient broth medium and then used as aliquots of fresh culture for further steps. The commercially available media, nutrient broth supplemented with 10 µg/mL sodium selenite, were used for all strains of inoculation and incubated at 39 °C (ADS1, ADS2) and room temperature ranged between 28 and 32 °C in (ADS18) for 24 h. Each inoculum contained 1 × 10^6^ of isolated bacterial cells. The culture was centrifuged at 6000 rpm for 15 min to harvest the bacterial pellets enriched with Se and then washed two times using deionized water to remove inorganic selenium which might be adsorbed in the bacterial cells [12]. Selenium-enriched bacterial cells were collected and lyophilized at −20 °C.

Furthermore, the collected bacterial biomass was subjected to ultrasonication to disrupt the bacterial cell walls and release their organic Se-content, while sonication was performed using ice water bath for 90 cycles, with 5 s on and 5 s off. The extraction of selenoprotein from Se-enriched bacterial cells was carried out using dialysis technique. The dialysis process was performed using dialysis sacks of flat width 25 mm, 12,000 Da, (Sigma-Aldrich, St. Louis, MO, USA) against deionized water, which was changed every 12 h for a total of 96 h to separate Se bounding protein [12]. The content in the dialysis tube was lyophilized and then used as source of bacterial selenoproteins.

### 2.2. Experimental Animals and Design

The animal care and management were conducted according to guidelines of Universiti Putra Malaysia (UPM) research policy. A total of 180 day-old female broiler chicks (Cobb 500) were obtained from a local commercial hatchery and kept in battery cages. Upon arrival, chicks were wing-banded individually, weighed, and randomly assigned to five treatment groups according to complete randomized design (CRD). Each group contained 36 chicks distributed among six cages, and each cage contained six chicks. Feed and water were made available ad libitum. All chicks received vaccinations against Newcastle disease (ND) and bronchitis (IB) on the seventh day. The vaccine of infectious bursal disease was applied on the 14th day through the intraocular route. A basal starter and finisher diets were prepared based on the broiler nutrient requirements (Table 1). The starter diet was provided to the chicks from 0 to 21 days while the finisher diet offered from day 22 to day 42 of age. The experimental groups involved T1, (basal diet); T2, (basal diet + 0.3 mg/kg feed Na2SeO3); T3, (Basal diet + 0.3 mg/kg feed ADS1 Se); T4, (Basal diet + 0.3 mg/kg feed ADS2 Se); T5, (Basal diet + 0.3 mg/kg feed ADS18 Se).

### 2.3. Breast Meat Sampling

When the experiment concluded at day 42, 12 birds from each dietary treatment (2 birds from each replicate) were selected randomly, weighed, and then scarified. The right half of breast meat was collected directly into plastic bag and kept at −80 °C for further analysis.

### 2.4. Carcass Characteristics

To determine the carcass characteristics, two birds per replicate was chosen, slaughtered, de-feathered, and eviscerated. The carcass weight and dressing percentage (carcass weight as a percentage of final BW) were recorded. Internal organs (liver, heart, spleen, and gizzard), breast meat, thigh, and drumstick were weighed and presented as a percentage of the birds live body weight.

### 2.5. Assay of Se Content in Breast Meat

Firstly, 0.5 g of meat samples were processed via microwave digestion system, by adding 5 mL of HNO_3_ and 1 mL H_2_O_2_ to the meat samples in a digestion cylinder. Then to obtain a 10 mL of working solution, around 9 mL of deionized water was added to the residual 1 mL of the digests. The Se level was determined directly in the resulted solution using the inductively coupled plasma mass-spectrometer (ICPMS) (Agilent Technologies, California, USA) following the process described by Wahlen et al. [13].

### 2.6. Determination of Meat Quality

#### 2.6.1. Drip Loss

A weight of 30 g of fresh breast meat sample was collected and weighed individually (as W1). Then the samples were vacuum-packed in sealed polyethylene plastic bags and kept in a chiller at 4 °C for 24 h, after which the samples taken out of the bags and dried gently, then weighed (as W2). The difference of sample weight was computed and presented as the percentage of the (W1) [14].

Drip loss (%) = ((W1 − W2)/W1) × 100(1)

#### 2.6.2. Cooking Loss

Cooking losses of the breast meat were determined after 20 h of slaughter according to the method of [15]. Samples were thawed overnight at 4 °C, the initial weight (W1) was recorded individually, and then all samples cooked at 80 °C for 20 min using a water bath. After cooking, all the breast meat samples were cooled at room temperature and then weighed as (W2). Cooking losses were considered as the difference between the initial (W1) and the final (W2) weight.

Cooking loss (%) = ((W1 − W2)/W1) × 100(2)

#### 2.6.3. Tenderness Measurement

Meat texture was assessed with the texture analyzer (TAHD plus^®^, Stable Micro System, Surrey, UK). Firstly, cooked samples were cut into sub-samples and were sheared perpendicular to the longitudinal route of the fibers [16]. Then, the samples’ shear forces were noted as the average of all sub-samples’ values, and the results were expressed in kilograms.

#### 2.6.4. Color Measurement

Breast meat samples were taken out from the freezing (−80 °C) and then kept at 4 °C overnight for thawing. The plastic packaging was released from the samples and then all the samples left to bloom in the air for 20 min before measuring the color by MINOLTA CR300 (Minolta Camera Co. Ltd., Osaka, Japan) [17].

#### 2.6.5. pH Measurement

The pH was measured using portable pH meter of (Mettler Toledo, AG 8603, Schwerzenbach, Switzerland). The instrument was calibrated before and immediately after each session according to manufacturer’s instructions. Around 0.5 g of each sample was homogenized with 10 mL of 5 mM sodium iodoacetate, 150 mM KCl solution using a homogenizer (Wiggen Hauser^®^ D-500, Berlin, Germany) for 20 s to avoid further glycolysis. Then, the homogenate was used to measure a pH at 20 ± 1 °C using the pH meter electrode [18].

### 2.7. Determination of Antioxidant Enzyme Activity

Glutathione peroxidase activity (GSH-Px), Total antioxidant (TAC), catalase activity, superoxide dismutase activity (SOD), and the level of 2-thiobarbituric acid reactive substances (TBARS) were measured in the breast meat. Meat samples were homogenized firstly with TBS buffer on ice, then were centrifuged for 10 min at 3000× *g* and 4 °C. The resulting supernatant was gathered for antioxidant enzymes assessment using Bioassay Systems Commercial kit (Bioassay Systems, Hayward, CA, USA).

### 2.8. Statistical Analysis

Analysis of the data was done by a one-way ANOVA in a totally randomized design with the diet type as the independent variable using one-way ANOVA (SAS institute, Carry, NC, USA). The differences between means were measured using Duncan’s multiple-range test. The significance was considered at *p* ≤ 0.05.

## 3. Results

### 3.1. Carcass Characteristics and Internal Organs

The effects of inorganic and bacterial selenoprotein on broiler’s carcass characteristics and internal organs are presented in Table 2. Dietary Se sources, either of inorganic or bacterial source, did not significantly affect the live body weight, relative weight of carcass, breast muscle, thigh muscle, drumstick, and abdominal fat, as well as the internal organs weights in broiler chickens.

### 3.2. Meat Selenium Content

**Figure 1:**
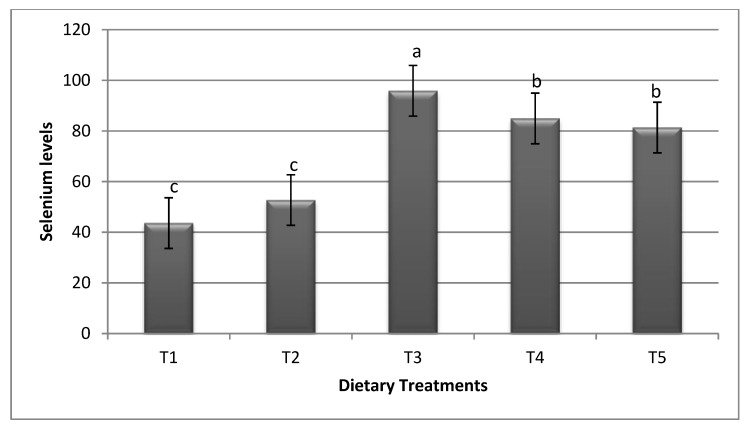
Effect of inorganic selenium and different sources of bacterial selenoprotein on breast meat selenium concentration (µg/kg). ^a,b,c^ Means in the same bar with different superscripts are significantly different (<0.05). T1: basal diet, T2: basal diet + 0.3 mg/kg feed sodium selenite, T3: basal diet + 0.3 mg/kg feed ADS1 Se, T4: basal diet + 0.3 mg/kg feed ADS2 Se, T5: basal diet + 0.3 mg/kg feed ADS18 Se.

Figure 1 indicates the results of the breast meat selenium content in broiler chickens fed bacterial selenoprotein and inorganic Se for 42 days. The present results show that bacterial selenoprotein raised the level of Se content significantly compared to inorganic Se and the basal diet, while there was no significant difference between sodium selenite and basal diet. The supplementation of bacterial selenoprotein (of ADS1) was associated with the highest breast meat Se content compared to ADS2 and ADS18, however, ADS2 and ADS18 were not significantly different.

### 3.3. Breast Meat Quality

The effects of dietary supplementation of organic and inorganic selenium sources on breast meat quality of broilers are presented in Table 3. Both supplemented Se sources (inorganic and bacterial selenoprotein) significantly (*p* < 0.05) influenced meat quality. Basal diet showed the highest percentage of drip loss, cooking loss, and shear force level; however, no significant effect was observed among Se-supplemented groups. Shear force was significantly lower in all bacterial selenoprotein than the basal diet (*p* < 0.001), but the inorganic Se did not differ significantly from the basal diet or the organic bacterial treatments.

The finding of pH and color values (L* (lightness), b* (yellow-green color), a* (red color)) of broiler’s breast muscle of different Se sources, showed that there was no significant difference among all experimental treatments in pH and color. These findings suggest that dietary Se of (bacterial selenoprotein or inorganic Se) had no effect on breast meat color and pH values.

### 3.4. Meat Antioxidant Capacity

**Figure 2:**
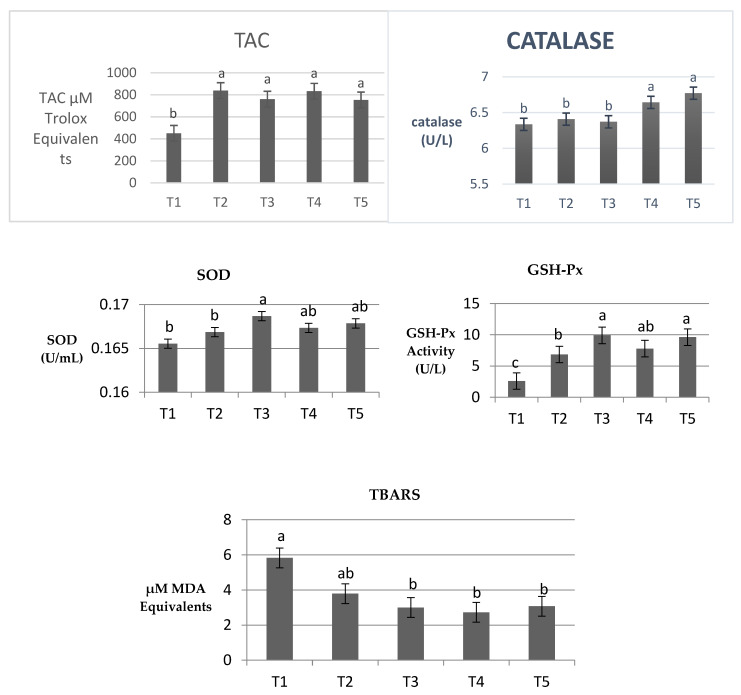
Effects of inorganic selenium and different sources of bacterial selenoprotein on meat antioxidant biomarkers. ^a,b,c^ Bars with no common letter differ significantly (*p* < 0.05). T1: basal diet, T2: basal diet + 0.3 mg/kg feed sodium selenite, T3: basal diet + 0.3 mg/kg feed ADS1 Se, T4: basal diet + 0.3 mg/kg feed ADS2 Se, T5: basal diet + 0.3 mg/kg feed ADS3 Se. TAC: total antioxidant capacity; SOD: superoxide dismutase; GPX: glutathione peroxidase.

Current study revealed that dietary bacterial selenoprotein supports the breast meat antioxidant status (Figure 2). Total antioxidant in breast meat showed significant improvement (*p* < 0.05) when Se supplemented the diets either in the inorganic or organic bacterial forms. However, bacterial selenoprotein of T4 and T5 indicated the highest catalase activity compared to the other treatments, with no significant differences between T1, T2, and T3. Bacterial selenoprotein (of ADS1) significantly increased SOD activity more than inorganic Se (*p* < 0.05), and the basal diet in the breast meat, bacterial selenoprotein of ADS2 and ADS18 had no significant effect compared to the other treatments. In addition to that, GSH-Px activity was significantly greater in all Se treatments compared to the basal diet (*p* < 0.001). However, T3 and T5 were significantly greater than T2 (*p* < 0.001), but no significant difference was found between T2 and T4. All bacterial selenoprotein sources reduced the TBARS level more than the basal diet with no significant differences compared to sodium selenite.

## 4. Discussion

The quality of carcass characteristics is influenced by the genetics and environmental factors including the nutritional aspect [19]. Dietary Se sources and levels did not affect broiler’s carcass characteristics [20]. Additionally, carcass traits and internal organs’ relative weight were not affected by the supplementation of different concentrations of organic Se to broiler chickens [21]. This supported our current results that indicated dietary inorganic and bacterial selenoprotein had no effect on the examined carcass characteristics and internal organs. However, according to Choct et al. [22], dietary organic Se (Se-yeast) increased eviscerated weight and breast yield, which was attributed to the possible ability of organic Se to enhance water retention and protein deposition in the tissues.

Dietary supplementation of different bacterial selenoprotein versus sodium selenite in broiler chickens significantly affects breast muscle Se content. All groups of bacterial selenoprotein accumulate more Se in the breast muscle with significant difference from inorganic Se and the basal diet, which may be attributed to the fact that bacterial Se-containing proteins such as (selenocysteine and selenomethionine) are capable to be accumulated in some tissues including kidney, liver [11], and meat. However, inorganic Se is absorbed less efficiently than organic form and excreted in the urine at a greater level due to the different metabolic pathways between both forms [23]. Previous data showed that dietary Se supplementation increased the Se concentration in the breast muscle, liver, and kidney in broiler chicken. However, the organic form of Se (Se yeast) showed more tissues Se content than sodium selenite [24,25]. Organic Se may have better bioavailability and is retained efficiently in the body than sodium selenite. Sodium selenite is absorbed less efficiently and excreted at a higher rate compared to organic Se [23].

The outcomes of this present study demonstrates that feeding of both Se sources, either inorganic or bacterial selenoprotein, had significant effects on drip loss, cooking loss, and shear force of chicken breast meat over 24 h of storage at 4 °C. Drip and cooking losses are considered useful indicators of the meat water holding capacity. Drip loss is affected by lipid peroxidation content and pH level [26]. Therefore, in this current study, the considerable decrease of TBARS levels of breast muscle in Se-supplemented groups may contribute to the considerably reduced drip loss and cooking loss. Our results were in agreement with Wang et al. [27] and Choct et al. [22], that Se supplementation reduced breast meat drip loss and cooking loss. However, there were no significant differences between sodium selenite and bacterial selenoprotein. Furthermore, according to Yang et al. [28], different Se sources had no significant effect on the shear force of broiler breast meat (*p* > 0.05). However, Cozzi et al. [29], reported that shear force of the meat of cattle was reduced significantly due to organic Se supplementation. In the present study, no significant difference between inorganic Se and bacterial organic source on the meat shear force was observed. While all Se-supplemented groups showed lower shear force than the basal diet, this may be due to breast meat WHC improvement. It has been well documented that, regardless of the amount of connective tissue of the muscles, WHC increases in raw and cooked meat, significantly affect cooked meat tenderness [30]. Moreover, in the current study, measurements of pH and color changes revealed that dietary Se supplementation of inorganic and bacterial organic forms did not significantly affect breast meat color and pH values. According to Perić et al. [2] and Yang et al. [28], supplementation of inorganic and selenoprotein in broiler chickens indicated no differences in breast meat’s pH and all values were within the normal range, while other studies conducted by Cai et al. [31] and Lisiak et al. [32], suggested that dietary Se supplementation had no effect on breast meat color. In contrast, Yang et al. [28], reported that supplementation of 0.3 ppm of organic Se in broiler chickens showed a significant increase in breast meat red color compared to inorganic Se. However, it is generally accepted that selenium is an important element for cellular antioxidant systems and the correlation between meat characteristics and oxidation resistance of breast muscle has been widely reported. In the present study, dietary bacterial Se as an organic source showed higher glutathione peroxidase activity in comparison with the basal diet; thus, drip loss and cooking loss reduction were observed, and consequently, tenderness was improved. These findings tend to suggest that all changes observed in the breast meat quality may be attributed to the direct effect of Se on antioxidant enzymes, and according to Juniper et al. [33], the excess Se concentration in the tissues, more than the requirements of antioxidant enzymes synthesis, may not show any effect on other meat quality indices.

In the current study, Se supplementation as inorganic or bacterial organic forms increased TAC, GSH-Px, and reduced TBARS level compared to the basal diet in the breast meat. However, bacterial selenoprotein showed a more superior effect than sodium selenite on GSH-Px, catalase, and SOD activity. It is well recognized that broiler meat contains high levels of poly-unsaturated fatty acids which increase their oxidation ability. Selenium plays an important role in controlling GSH pool, and it is abundant in selenoenzyme GSH-Px. Antioxidants such as GSH-Px, SOD, and CAT are considered the primary antioxidant defense against free radicals and they may reduce the oxidation damages [34]. The present finding is supported by Jiang et al. [35], who reported that organic Se in the form of selenomethionine improved the breast meat antioxidant status more than sodium selenite. Moreover, the supplementation of organic Se in the form of Se-yeast alone or in-combination with sodium selenite increased the activity of CAT, SOD, and TAC with MDA reduction compared to sodium selenite in broiler breast meat [36]. In addition, Wang et al. [18] suggested that organic Se improved the antioxidant status of broiler meat more than sodium selenite by increasing the antioxidant enzymes activity and reducing the lipid peroxidation end product.

Khan et al. [37] revealed that supplementation of Se-enriched probiotic improved breast meat quality and enhanced the antioxidant capacity by increasing GSH-Px and SOD activity with MDA reduction. Also, a significant reduction in breast muscle lipid peroxidation and drip loss was observed when Se was supplemented as a nano-Se form in broiler chickens [38]. However, CAT and SOD are not Se-dependent enzymes, but their activities may be influenced by dietary Se indirectly through the effect of Se on thyroid hormone metabolism. Thyroid hormones play important roles in lipid metabolism, and any possible high thyroxine production may stimulate the free radicals and then the antioxidant enzymes activities [36]. Therefore, these data suggest that bacterial selenoprotein improved the breast meat antioxidant status in broiler chickens and this may be attributed to the presence of Se-met and Se-cys in the bacterial Se [39], both selenoprotein sources are more bioavailable than inorganic Se (sodium selenite) and can improve the antioxidants capacity and decrease the production of free radicals.

## 5. Conclusions

Currently, we conclude that bacterial selenoprotein is more effective than sodium selenite for depositing Se and improving the antioxidant status in the breast meat of broiler chickens. Both inorganic Se and bacterial selenoprotein improved meat quality by reducing drip loss, cooking loss, and shear force of breast meat. Therefore, bacterial selenoprotein may act as an interesting source of organic Se for broilers to produce Se-enriched breast meat, and benefit the animals and also humans through the food chain.

## Figures and Tables

**Table 1:** Ingredients and nutrient content of the basal diet.

Ingredients	Starter %	Finisher %
Corn	52.5	56.250
Palm oil (Refine)	5.00	6.00
Soybean meal (44% Crude Protein)	32.50	30.00
Fish meal (58% Crude Protein))	5.15	3.25
L-Lysine	0.25	0.25
DL-Methionine	0.25	0.25
Dicalcium phosphate 18%	1.60	1.85
Calcium carbonate	0.60	0.35
Salt (Nacl)	0.30	0.30
Mineral premix ^a^	0.15	0.15
Vitamin premix ^b^	0.10	0.10
Toxin binder ^c^	0.15	0.15
Choline chloride	0.10	0.10
Wheat pollard	1.35	1.00
**Calculated nutrient content (g/kg DM)**		
ME (kcal/kg)	3081.1	3152.8
Crude protein	22.04	20.09
Crude fat	7.57	8.004
Calcium	1.189	1.0440
Phosphorus	0.786	0.768
Avail. P for poultry	0.472	0.450
Analyzed Se (mg/kg) ^d^	0.079	0.087

^a^ Mineral premix provided the following per kg diet: iron 120 mg, manganese 150 mg, copper 15 mg, zinc 120 mg, iodine 1.5 mg, and cobalt 0.4 mg; ^b^ Vitamin premix provided the following per kg diet: Vitamin A (retinyl acetate) 10.32 mg, cholecalciferol 0.250 mg, vitamin E (DL-tocopheryl acetate) 90 mg, vitamin K 6 mg, cobalamin 0.07 mg, thiamine 7 mg, riboflavin 22 mg, folic acid 3 mg, biotin 0.04 mg, pantothenic acid 35 mg, niacin 120 mg and pyridoxine 12 mg; ^c^ Toxin binder contains natural hydrated sodium calcium aluminum silicates to reduce the exposure of feed to mycotoxins; ^d^ The Se content was measured using Inductively Coupled Plasma Mass Spectrometry (ICP.MS); DM: Dry matter.

**Table 2:** Effects of inorganic selenium and different sources of bacterial selenoprotein on carcass characteristics and internal organs in broiler chickens

	Dietary Treatments ^1^						
Parameters	T1	T2	T3	T4	T5	SEM	*p*
**Carcass characteristics**							
Final body weight (g)	2008	2082.1	2054.8	2075.4	2093.9	21.65	0.064
Carcass yield%	72.39	72.36	73.44	75.85	76.57	1.039	0.141
Breast%	36.02	35.80	34.45	34.82	35.87	0.628	0.927
Thigh%	9.56	8.56	8.71	8.12	7.45	0.439	0.238
Drumstick%	7.91	5.90	5.80	5.96	6.26	0.421	0.074
Abdominal Fat%	1.296	1.827	2.482	2.327	2.554	0.167	0.060
**Internal Organs (% of live weight)**							
Liver%	2.827	2.849	2.817	2.395	2.877	0.088	0.412
Spleen%	0.164	0.159	0.173	0.138	0.154	0.008	0.788
Gizzard%	3.981	3.312	3.854	3.284	3.511	0.142	0.452
Thymus%	0.328	0.212	0.159	0.228	0.279	0.024	0.268
Bursal%	0.067	0.088	0.099	0.069	0.068	0.007	0.657

^1^ T1: basal diet, T2: basal diet + 0.3 mg/kg feed sodium selenite, T3: basal diet + 0.3 mg/kg feed ADS1 Se, T4: basal diet + 0.3 mg/kg feed ADS2 Se, T5: basal diet + 0.3 mg/kg feed ADS18 Se; SEM = standard error of mean.

**Table 3:** Effects of inorganic selenium and different sources of bacterial selenoprotein on meat drip loss, cooking loss, shear force, color, and meat pH in broiler chickens.

Parameters	Dietary Treatments ^1^						
	T1	T2	T3	T4	T5	SEM	*p*
Drip loss %	1.96 ^a^	1.44 ^b^	1.42 ^b^	1.32 ^b^	1.49 ^b^	0.082	0.0311
Cooking loss %	25.62 ^a^	24.07 ^b^	24.40 ^b^	23.25 ^b^	23.23 ^b^	0.290	0.0220
Shear force (kg)	1.16 ^a^	0.976 ^ab^	0.856 ^b^	0.892 ^b^	0.915 ^b^	0.54	0.0001
Color							
L*	54.65	55.33	54.41	53.66	55.05	0.304	0.539
a*	5.08	5.61	4.14	5.75	5.63	0.407	0.500
b*	15.05	15.49	14.67	15.26	15.12	0.307	0.061
pH	6.18	6.01	6.06	6.03	6.09	0.029	0.053

^1^ T1: basal diet, T2: basal diet + 0.3 mg/kg feed sodium selenite, T3: basal diet + 0.3 mg/kg feed ADS1 Se, T4: basal diet + 0.3 mg/kg feed ADS2 Se, T5: basal diet + 0.3 mg/kg feed ADS18 Se; L* (lightness); b* (yellow-green color); a* (red color); ^a,b^ Means in the same row with different superscripts are significantly different; SEM = standard error of mean.
